# Oat bran and wheat bran impact net energy by shaping microbial communities and fermentation products in pigs fed diets with or without xylanase

**DOI:** 10.1186/s40104-020-00505-7

**Published:** 2020-10-08

**Authors:** Zhiqian Lyu, Li Wang, Jinrong Wang, Zhenyu Wang, Shuai Zhang, Junjun Wang, Jinlong Cheng, Changhua Lai

**Affiliations:** 1 State Key Laboratory of Animal Nutrition, College of Animal Science and Technology, China Agricultural University, No. 2 Yuanmingyuan West Road, Beijing, 100193 China; 2 INRA-Agrocampus, UMR PEGASE1348, Saint-Gilles, 35000 Rennes, France; 3 College of Bioengineering, Henan University of Technology, Zhengzhou, 450001 China; 4 Jiangsu Youshi Biotechnology Development Co. Ltd., Suqian, 223800 Jiangsu China

**Keywords:** Bacterial community, Bacterial metabolites, Dietary fiber, Exogenous enzyme, Net energy, Pig

## Abstract

**Background:**

Dietary fiber can be fermented in gut of pigs and the end products of fermentation were short-chain fatty acids (SCFA). The SCFA had positive effects on gut bacteria and host immune system. In addition, SCFA can provide a part of available energy for pigs. However, there were limited reports on the relationship between dietary fiber, gut bacteria, and energy metabolism. Therefore, this study investigated how dietary fiber and enzyme addition impacted energy metabolism by acting on the microbial community and SCFA.

**Methods:**

Wheat bran (WB) was added to the corn-soybean meal-based diet at the levels of 12% and 27%, and oat bran (OB) at 15% and 36%. One of each diet was supplemented with or without 5000 U/kg feed of xylanase, so a total of 10 diets were allotted to 60 growing pigs (initial body weight: 27.2 ± 1.2 kg) using a randomized complete block design. The experiment was conducted in 10 consecutive periods using 6 similar open-circuit respiration chambers. Each pig was used for one 20-day period. During each period, six pigs were allowed 14 d to adapt to the diets in metabolic cages followed by 6 d (from d 15 to d 20) in respiration chambers to measure heat production (HP).

**Results:**

Pigs fed 36% OB diets had greater (*P* <  0.05) nutrient digestibility and net energy (NE) values compared to those fed 27% WB diets. Apparent digestibility coefficients of dry matter (DM) and crude protein (CP) were lower (*P* < 0.05) in pigs fed 27% WB diets compared with those fed 12% WB diets. Enzyme addition improved (*P* < 0.05) the NE values (11.37 vs. 12.43 MJ/kg DM) in diets with 27% WB. Supplementation of xylanase did not affect NE values for basal diets, OB diets and 12%WB diets. Compared with diets with 36% OB, pigs fed 27% WB-based diets excreted more total SCFA, acetate and propionate (expressed as g/kg feed DM) in fecal samples of pigs (*P* < 0.05). Pigs in the WB diets had greater proportion of phylum Bacteroidetes while phylum Firmicutes were greater in pigs fed OB diets (*P* < 0.05). Pigs fed WB diets had greater (*P* < 0.05) abundance of Succinivibrio and *Prevotella,* which were associated with fiber degradation and SCFA production.

**Conclusion:**

Our results indicated diets supplied by high level of OB or WB promote the growth of fiber-degrading bacteria. The differences in fiber composition between WB and OB led to differences in nutrient digestibility and bacterial communities, which were ultimately reflected in energy metabolism. Enzyme supplementation improved nutrient digestibility as well as NE values for 27% WB diets but not for other diets, which indicated that effects of enzyme were related to type and level of dietary fiber in diets.

## Background

Dietary fiber (DF) means carbohydrate polymers with ten or more monomeric units which are not hydrolyzed by the endogenous enzymes in the small intestine of humans [1]. The microbial conversions of DF to monosaccharides in the gut involves a number of principal reactions mediated by the carbohydrate-active enzymes. These enzymes are from specific members of the gut microbiota and have the function to cleave the glycosidic bonds between sugar monomers or between carbohydrate and non-carbohydrate structures. Major end products from these fermentations are the short-chain fatty acids (SCFA) [2], mainly acetate, propionate, and butyrate. Recent studies show that SCFA from microbial fermentation generates 11% to 24% of the total digestible energy (DE) as wheat bran (WB) level increases from 10% to 30% when fed to pigs [3]. It suggests that DF has a final impact on energy metabolism by shaping microbes and regulating SCFA production [4]. However, there is little information on the relationship between DF, gut bacteria, SCFA, and energy metabolism [5].

Oat bran (OB) and WB are rich in DF that has negative effects on nutrient digestibility due to their anti-nutritive properties, but they may act as substances and energy sources to stimulate the intestinal microbiota [6]. Dietary fiber in OB is easier fermentable energy source for bacteria in the large intestine due to their lower proportion of lignified fiber types and higher proportion of soluble fiber types compared with fiber in WB. Specially, OB is rich in mixed linkages β-glucan which to a high degree is soluble and insoluble arabinoxylan [7]. By contrast, WB contained rich insoluble arabinoxylan. In the small intestine, β-glucan in OB is partly digested while arabinoxylan in OB or WB is hardly digested [7, 8]. Accordingly, arabinoxylan is considered as one of the main components for DF in OB and WB to resist fermentation by bacteria in gastrointestinal tract. By adding xylanase to feeds, xylose backbone of arabinoxylan can be cleaved, thereby improving accessibility and utilization of nutrients in WB or OB diets and providing some fermentable oligosaccharides degraded from indigestible polysaccharides to intestinal bacteria [9]. Substrates available to intestinal bacteria and physiology of lumen change along gastrointestinal tract, and consequently intestinal bacteria are altered. Therefore, DF and exogenous enzymes, independently or in combination interaction are important means of regulating intestinal bacteria and ultimately affecting energy metabolism [4–6].

Therefore, we hypothesize that the diets supplemented with different fiber sources, with or without xylanase, could exert different impacts on nutrient digestibility and NE values of diets, and these differences were related to the activity of certain bacteria. Specially, our objectives are to determine effects of dietary characteristics and xylanase addition on nutrient digestibility, NE values of diets, fecal SCFA concentrations, and fecal bacterial communities of growing pigs.

## Methods

### Experimental diets and enzyme

As shown in Table 1, WB diets contained 12% or 27% WB which was added at the expense of corn and soybean meal. Oat bran diets contained 15% or 36% OB which was added at the expense of corn and soybean meal. Enzyme was added to one half of each diet at the rate of 40 g/1000 kg diet to supply enzyme activity of 5000 U/kg complete feed (as-fed basis; Table 1**)**. The enzyme was first mixed in the vitamin-mineral premix and then mixed with other ingredients. A total of 10 diets were fed to 60 growing pigs with a randomized complete block design. Diets were formulated on NE basis to meet estimated AA requirements for growing pigs [10]. Diets were formulated based on total dietary fiber (TDF) content in OB and WB. Specially, DF content (calculated values) between 36% OB diets and 27% WB diets or between 15% OB diets and 12% WB diets was similar. The xylanase used in the current study was endo-1, 4-β-xylanase in granular form. The xylanase was provided by Bestzyme Co. Ltd. (Jinan, China) with enzyme activity of 200,000 U/g.

**Table 1:** Ingredients of experimental diets (as-fed basis)^1^

Item		Test diets			
	Basal diet	36% OB	27% WB	15% OB	12% WB
Ingredients, %					
Corn	71.60	45.12	51.74	60.57	62.77
Soybean meal	24.96	15.73	18.04	21.11	21.88
Oat bran	–	36.00	–	15.00	–
Wheat bran	–	–	27.00	–	12.00
Dicalcium phosphate	0.90	0.90	0.90	0.90	0.90
Limestone	0.90	0.90	0.90	0.90	0.90
Salt	0.35	0.35	0.35	0.35	0.35
Premix^2^	0.50	0.50	0.50	0.50	0.50
Lys HCl	0.50	0.32	0.36	0.42	0.44
Met	0.07	0.04	0.05	0.06	0.06
Thr	0.16	0.10	0.12	0.14	0.14
Val	0.06	0.04	0.04	0.05	0.05

^**1**^*OB* Oat bran, *WB* Wheat bran. The xylanase was provided by Bestzyme Co. Ltd. (Jinan, China). Enzyme was added to one half of each diet at the rate of 40 g/1000 kg diet to supply enzyme activity of 5000 U/kg complete feed (as-fed basis)

^2^Vitamin-mineral premix supplied the following per kg of complete diet: vitamin A as retinyl acetate, 5512 IU; vitamin D_3_ as cholecalciferol, 2200 IU; vitamin E as *DL*-alpha-tocopheryl acetate, 30 IU; vitamin K_3_ as menadione nicotinamide bisulfite, 2.2 mg; vitamin B_12_, 27.6 μg; riboflavin, 4 mg; pantothenic acid as *DL*-calcium pantothenate, 14 mg; niacin, 30 mg; choline chloride, 400 mg; folacin, 0.7 mg; thiamin as thiamine mononitrate, 1.5 mg; pyridoxine as pyridoxine hydrochloride, 3 mg; biotin, 44 μg; Mn as MnO, 40 mg; Fe as FeSO_4_·H_2_O, 75 mg; Zn as ZnO, 75 mg; Cu as CuSO_4_·5H_2_O, 25 mg; I as KI, 0.3 mg; Se as Na_2_SeO_3_, 0.3 mg

### Animals and experimental procedure

The animal procedures used in this experiment, including animal care and use, were approved by the Animal Care and Use Ethics Committee of China Agricultural University (Beijing, China). Sixty growing barrows (initial BW of 27.2 ± 1.2 kg) were allotted to 10 diets with 6 replicate pigs per diet in a randomized block design. The experiment was conducted in 10 consecutive periods using 6 similar open-circuit respiration chambers as described by Lyu et al. [11]. Each pig was used for one 20-d period. During each period, 6 pigs were allowed 14 d to adapt to diets in metabolic cages followed by 6 d in respiration chambers to measure heat production (HP). Pigs were fed their assigned diets at 2.3 MJ ME/(kg BW^0.6^·d) on basis of BW measured on d 0, 7, and 14 [12]. Pigs were fed equal-sized dried meals twice daily at 08:30 and 15:30 h in mash form and had free access to water via a low-pressure nipple drinker. Urine was collected in buckets containing 50 mL of 6 mol/L HCl. From d 15 to 19 of each period, total feces and urine were collected from the chambers once daily at 8:30 h and then stored at − 20 °C. On d 19 (from 7:30  to 9:00 h) of each period, six freshly voided fecal samples from each dietary treatment were collected to measure SCFA concentrations and bacterial community. Fecal samples were acquired using a 5-mL centrifuge tube, put in liquid nitrogen and then were stored at − 80 °C. Diet samples were collected in each collection period and then stored at − 20 °C. At the end of experiment, diet samples were mixed and subsampled based on treatments to measure xylanase activity. From d 19 to 20 of each period, pigs were fasted.

The design of 6 open-circuit respiration chambers were reported by van Milgen et al. [13] and Lyu et al. [11]. Volume of each chamber was approximately 7.8 m^3^, and relative humidity in chamber was controlled at 70%. During the feeding period, the temperature was 22 °C, and it was increased to 24 °C on the fasted day. The measurement range of analyzers was 19.5% to 21% for O_2_, 0 to 1% for CO_2_, 0 to 0.1% for CH_4_, and 0 to 0.1% for NH_3_ with a sensitivity of 0.2% within the measurement range.

### Sample preparation and chemical analyses

Diets were collected during each feeding period to measure chemical composition. Fecal samples were oven-dried for 72 h at 65 °C and were ground through a 1-mm screen for further chemical analysis. Urine samples were pooled separately within pig.

Xylanase activity in diets was analyzed using spectrophotometric method as described by Lærke et al. [14]. The chemical analyses of ingredients and diets included dry matter (DM, method 930.15) [15], crude protein (CP, method 984.13) [15], ether extract (EE) [16], ash (method 942.05) [15], Ca (method 968.08) [15], and P (method 946.06) [15]. Gross energy (GE) of ingredients, diets, feces, and urine (UE) was determined using an isoperibol calorimeter (Parr 6300 Calorimeter, Moline, IL USA). The neutral detergent fiber (NDF) and acid detergent fiber (ADF) in ingredients, diets, and feces were determined using a fiber analyzer (model A220 fiber analyzer; Ankom Technology, Macedon, NY, USA) following a procedure by Van Soest et al. [17]. The combination of enzymatic and gravimetric procedures reported by Prosky et al. [18] was used to measure TDF, insoluble dietary fiber (IDF) and soluble dietary fiber (SDF) in ingredients and diets. Non-starch polysaccharides (NSP) and their constituent sugars in OB and WB were determined based on alditol acetates by gas-liquid chromatography (Aglilent GC 6890, USA) with a flow of 20 mL/min and split 40:1. The column temperature was 220 °C and the injector and detector temperature were 250 °C.

Fecal SCFA concentrations were measured using the method reported by Liu et al. [19]. About 0.5 g of fecal sample was placed in a centrifuge tube with 8 mL of distilled water. The mixture was centrifuged at 13,000 r/min for 10 min to obtain the supernatant. One milliliter of supernatant was diluted 1:50 with water and filtered through a 0.22-mm nylon membrane filter. Filtered supernatant was analyzed using high-performance ion exchange chromatograph (ICS 3000 Dionex, USA).

### Analysis for bacterial microbiota by 16S RNA sequences

Bacterial DNA was extracted from fecal samples using the DNA Kit (Omega Bio-tek, Norcross, GA, USA). NanoDrop 2000 UV-VIS spectrophotometer (Thermo Scientific, Wilmington, USA) was used to determine DNA concentration and purification. Quality of DNA was assessed by 1% agarose gel electrophoresis. The V3-V4 hypervariable regions of the bacteria 16S rRNA gene were amplified with primers 338F (5′-ACTCCTACGGGAGGCAGCAG-3′) and 806R (5′-GGACTACHVGGGTWTCTAAT-3′) by thermocycler PCR system (GeneAmp 9700, ABI, USA). The PCR products were extracted from a 2% agarose gel and then purified using the AxyPrep DNA Gel Extraction Kit (Axygen Biosciences, Union City, CA, USA) and quantified using QuantiFluor™-ST (Promega, USA).

Pooled and purified amplicons in equimolar and paired-end were sequenced (2 × 300 bp) on an Illumina MiSeq platform (Illumina, San Diego,USA) according to the standard protocols introduced by Majorbio Bio-Pharm Technology Co. Ltd. (Shanghai, China). Raw fastq files were quality-filtered by Trimmomatic and merged by FLASH.

Operational taxonomic units (OTU) were clustered with 0.97 similarity cutoff using UPARSE (version 7.1 http://drive5.com/uparse/). The taxonomy of each 16S rRNA gene sequence was analyzed by Ribosomal Database Project (RDP) Classifier algorithm (http://rdp.cme.msu.edu/) against the Silva (SSU123) 16S rRNA database using confidence threshold of 0.70.

### Calculations

The apparent total tract digestibility (ATTD) of nutrients, total heat production (THP), retained energy (RE), and NE values in diets were calculated using the following equations [12, 20]: ATTD = [(Fi − Ff)/Fi] × 100%, where Fi was total intake of energy (kJ) or nutrients (g), and Ff was the total fecal output of energy (kJ) or nutrients (g); THP = 16.18 × O_2_ + 5.02 × CO_2_–2.17 × CH_4_–5.99 × urinary nitrogen excretion, where THP was in kilojoule, O_2_ was oxygen consumption in liters, CO_2_ was carbon dioxide production in liters, and CH_4_ was methane production in liters. Urinary nitrogen excretion was in grams. The data collection for O_2_, CO_2_, and CH_4_ used the standard procedure as described by Lyu et al. [11]. In addition, to eliminate the effects of activity of pigs, FHP was calculated using the equation for THP with gas data obtained from 22:30 h (d 19) to 06:30 h (d 20). To calculate the FHP throughout the day, the 8-h FHP was extrapolated to a 24-h period; RE = (ME intake – THP), where RE was in kilojoule, and ME intake and THP were in kilojoule per day. The retained energy as protein (RE_P_) was calculated based a recognized formula (nitrogen × 6.25 × 23.84, kJ/g), whereas RE as lipids (RE_L_) was calculated as the difference between RE and RE_P_ [21]; NE = (RE + FHP)/DM intake, where NE was in kilojoule per kilogram DM; RE and FHP were in kilojoule per day and DM intake was in kilograms per day. The DE of diets was calculated by subtracting energy lost in the feces from GE. The ME of diets was calculated by subtracting energy lost in the urine and methane from DE. Energy lost as methane (CH_4_E) was calculated assuming 1 L of CH_4_ contained 39.54 kJ energy [20]. The DE, metabolizable energy (ME) and NE content of OB and WB in diets containing 36% OB and 27% WB were calculated using the difference method, respectively [22].

### Statistical analysis

All data of the experiment were averaged per pig and were analyzed using the MIXED procedure of SAS (SAS Institute Inc., Cary, NC, USA). The experimental unit was the individual pig. Diet, enzyme and their interactions were treated as the fixed effects and collection, period and chamber as random effects. The LSMEANS statement with Tukey’s adjustment was used to separate mean values. To eliminate the effect of ME intake, the THP and RE data were adjusted for each collection period by covariance analysis for ME intake of 2322 kJ/(kg BW^0.60^·d) (mean value for the experiment). A mixture of two fecal samples was used to analyze bacterial communities. Bacterial diversity was analyzed using standardized OTU reads with the help of R software. Relative abundance of bacteria at phylum and family levels were showed as bar plots. Heatmap was used to analyze bacterial communities at genus level. The linear discriminant analysis (LDA) effect size (LEfSe) algorithm was adopted to classify abundances of differential bacteria from phylum to genus. The logarithmic LDA value of 2.0 was used to be the criterion. The comparative analysis between OB-based diets (*n* = 12) and WB-based (*n* = 12) diets or between high-fiber diets (36% OB diets and 27% WB diets; *n* = 12) and low-fiber diets (15% OB diets and 12% WB diets; *n* = 12), was conducted based on the method of Welch’s t-test. If *P* < 0.05, it was considered significant difference. The contrast for data obtained in the enzyme-supplemented group to those obtained in enzyme-free group was also calculated using same method to validate the enzyme effect.

## Results

### Chemical composition of ingredients and diets

Wheat bran contained more content of NDF, ADF, hemicellulose, and IDF but lower content of SDF than OB (Supplementary Table S1). For NSP composition, OB contained more soluble NSP (7.8% vs. 3.9%), especially glucose. By contrast, WB contained more insoluble NSP (28.7% vs. 15.6%), mainly arabinose and xylose. In addition, OB contained more content of CP (22.0% vs. 15.1%), starch (28.1% vs. 8.2%) and EE (7.6% vs. 3.2%) than WB. Dietary fiber content was similar between 36% OB diets and 27% WB diets or between 15% OB diets and 12% WB diets, which was consistent to the study design (Table 1 and Table 2).

**Table 2:** Chemical composition of experimental diets (DM basis)^1^

Item^2^	Basal diet		36% OB		27% WB		15% OB		12% WB	
	–	+	–	+	–	+	–	+	–	+
Analyzed composition										
GE, MJ/kg DM	18.21	18.37	19.00	19.10	18.62	18.65	18.66	18.58	18.60	18.45
CP, % DM	19.5	19.7	21.1	21.6	18.5	18.9	20.3	20.4	19.8	19.0
Ether extract, % DM	3.6	3.5	5.9	5.6	3.8	4.1	4.6	4.8	3.9	4.0
NDF, % DM	10.3	10.9	17.9	15.7	19.4	19.6	12.1	11.6	14.2	14.5
ADF, % DM	3.9	4.1	5.3	5.1	6.1	6.4	4.4	4.2	5.0	5.0
TDF, % DM	14.9	14.6	23.6	24.3	24.1	23.7	18.4	19.0	18.9	18.3
SDF, % DM	2.9	2.7	7.8	7.7	2.8	2.9	4.9	5.0	2.8	2.5
IDF, % DM	12.0	11.9	15.8	16.6	21.3	20.7	13.6	14.1	16.1	15.8
Ash, % DM	4.8	4.9	5.5	6.0	5.6	5.6	5.1	5.2	5.1	5.3
Enzyme activity, U/kg	–	5325	–	5120	–	4876	–	4795	–	5145

^1^Data are the means of two replicates of analyzed values

^**2**^*OB* Oat bran; *WB* Wheat bran; *GE* Gross energy; *DM* Dry matter; *CP* Crude protein; *NDF* Neutral detergent fiber; *ADF* Acid detergent fiber; *TDF* Total dietary fiber; *SDF* Soluble dietary fiber; *IDF* Insoluble dietary fiber. The – and + symbol represents enzyme-free diets and enzyme addition diets, respectively

### Nutrient digestibility, nitrogen balance, and energy metabolism

There were interactions (*P* < 0.05) between diet and enzyme supplementation on ATTD of DM, GE, CP and ADF (Table 3). In the enzyme-free treatments, 27% WB diets had lower (*P* < 0.05) ATTD of DM, GE, CP, ADF and OM than OB diets and basal diets. Pigs fed 27% WB diets had lower (*P* < 0.05) ATTD of DM, CP and ADF than those fed 12% WB diets. By contrast, there were no differences on the ATTD of nutrients between 36% OB diets and 15% OB diets. The nitrogen output from feces was greater (*P* < 0.05) in 27% WB diets than that in basal diets and 15% OB diets. Total nitrogen retention was not affected by dietary characteristics. Supplementation of enzyme improved (*P <* 0.05) ATTD of DM, CP and ADF in 27% WB diets, and no differences were observed for the enzyme effects in other dietary treatments.

**Table 3:** Effect of dietary characteristics and xylanase addition on nutrient digestibility and nitrogen balance of growing pigs (DM basis)^1^

Item	Xylanase-free diets					Xylanase supplementation diets					SEM	*P*-value		
	Basal diet	36% OB	27% WB	15% OB	12% WB	Basal diet	36% OB	27% WB	15% OB	12% WB		Diet	Xylanase	D×X^2^
Average BW	35.0	35.5	35.0	35.6	36.5	36.6	38.2	35.9	35.1	35.6	0.96	0.572	0.212	0.313
DMI, kg/d	1.21	1.23	1.28	1.25	1.28	1.26	1.37	1.34	1.25	1.28	0.04	0.295	0.051	0.421
ATTD, %														
DM	91.0^a^	86.8^bc^	80.7^d^	88.8^abc^	85.2^c^	89.9^ab^	86.0^bc^	85.3^c^	89.4^abc^	87.2^abc^	1.00	< 0.001	0.043	0.041
GE	90.2^a^	86.0^b^	79.6^c^	87.9^ab^	84.2^bc^	89.1^ab^	85.4^b^	81.6^bc^	88.4^ab^	86.4^ab^	1.08	< 0.001	0.082	0.048
CP	89.6^a^	86.9^a^	80.2^c^	88.2^a^	84.8^ab^	88.5^a^	84.7^ab^	85.7^ab^	89.0^a^	87.0^a^	1.44	< 0.001	0.045	0.043
NDF	63.5^ab^	70.0^a^	53.3^b^	63.4^ab^	57.5^ab^	61.0^ab^	63.5^ab^	58.9^ab^	65.2^ab^	60.7^ab^	2.74	0.003	0.861	0.215
ADF	65.2^a^	58.1^ab^	44.9^c^	61.4^a^	56.2^ab^	63.8^a^	53.7^ab^	53.5^ab^	62.1^a^	60.4^a^	3.29	0.022	0.025	0.036
OM	92.1^a^	88.8^ab^	82.6^c^	90.3^ab^	86.7^bc^	91.1^a^	88.1^ab^	86.6^bc^	90.8^ab^	88.5^b^	0.93	< 0.001	0.122	0.064
UE/DE	1.80	1.97	2.47	1.85	1.77	1.54	2.02	1.63	1.47	1.54	0.26	0.412	0.118	0.353
CH_4_E/DE	0.58	0.40	0.57	0.52	0.45	0.51	0.31	0.34	0.55	0.51	0.08	0.142	0.243	0.385
Nitrogen balance, g/d														
Intake	36.8^b^	40.4^ab^	40.1^ab^	39.0^b^	39.3^ab^	38.3^b^	45.0^a^	41.7^ab^	39.0^b^	39.1^b^	1.27	0.002	0.075	0.353
FN	4.0^c^	5.4^abc^	7.7^a^	4.8^bc^	6.0^abc^	4.7^bc^	7.1^ab^	7.2^ab^	4.5^bc^	5.2^abc^	0.58	< 0.001	0.702	0.181
UN	7.1	7.0	6.3	7.7	6.5	6.2	7.6	7.0	7.7	8.7	0.80	0.544	0.298	0.395
RN	25.6	28.0	26.1	26.5	26.8	27.5	30.3	27.5	26.7	25.2	1.28	0.324	0.142	0.571

^a,b,c,d^Values with different characters in superscripts were different (*P* < 0.05) in the same row

^1^Treatment means are reported as least squares means and are based on 6 observations per treatment. *OB* Oat bran, *WB* Wheat bran; *BW* Body weight (mean values in collection period); *DM* Dry matter; *DMI* Dry matter intake; *ATTD* Apparent total tract digestibility; *GE* Gross energy; *CP* Crude protein; *NDF* Neutral dietary fiber; *ADF* Acid detergent fiber; *OM* Organic matter; *UE* Urinary energy; *CH4E* Methane energy; *FN* Nitrogen output from feces; *UN* Urinary nitrogen excretion; *RN* Total retained nitrogen; *SEM* Standard error of the mean

^2^D×X is the interaction between diet and xylanase supplementation

No interactions between diet and enzyme on the energy retention were found (Supplementary Table S2). The THP, FHP, and RE data were not affected by dietary characteristics. Enzyme supplementation improved (*P* < 0.05) ME intake in pigs. There were interactions (*P* < 0.05) between diet and enzyme supplementation on ME and NE content of diets (Table 4). The NE:ME ratios and ME:DE ratios were not affected by diets and enzyme supplementation. The DE content was lower (*P* < 0.05) in pigs fed WB-based diets compared with those fed OB-based diets and basal diets. In enzyme-free dietary groups, 27% WB diets had lower (*P* < 0.05) ME and NE values than other diets. There were no differences for the ME and NE values between 36% OB diets and 12% OB diets. Enzyme addition improved the NE values (*P* < 0.05) in pigs fed 27% WB diets. Enzyme addition improved (*P* <  0.05) ME and NE content of WB from 11.75 to 13.02, and 8.77 to 10.04 (MJ/kg of feed DM) (Table 5).

**Table 4:** Effect of dietary characteristics and enzyme addition on energy utilization and energy values of experimental diets (DM basis)^1^

Item	Xylanase-free diets					Xylanase supplementation diets					SEM	*P*-value		
	Basal diet	36% OB	27% WB	15% OB	12% WB	Basal diet	36% OB	27% WB	15% OB	12% WB		Diet	Xylanase	D×X^2^
Energy utilization, %														
ME/DE	97.6	97.6	96.9	97.6	97.7	97.9	97.7	98.0	98.0	97.6	0.28	0.801	0.073	0.241
NE/ME	82.8	82.7	79.2	82.2	81.5	83.4	81.6	83.3	81.7	82.6	1.56	0.924	0.186	0.709
Energy values, MJ/kg DM														
DE	16.40^a^	16.38^a^	14.81^c^	16.43^a^	15.55^bc^	16.36^a^	16.32^a^	15.22^c^	16.40^a^	16.07^ab^	0.16	< 0.001	0.130	0.232
ME	16.01^a^	15.99^a^	14.35^d^	16.04^a^	15.20^bc^	16.02^a^	15.94^ab^	14.92^c^	16.07^a^	15.68^abc^	0.16	< 0.001	0.049	0.034
NE	13.26^a^	13.23^a^	11.37^c^	13.19^a^	12.38^b^	13.36^a^	13.00^a^	12.43^b^	13.13^a^	12.95^ab^	0.27	< 0.001	0.043	0.041

^a,b,c^Values with different characters in superscripts were different (*P* < 0.05) in the same row

^1^Treatment means are reported as least squares means and are based on 6 observations per treatment. *OB* Oat bran, *WB* Wheat bran; *DE* Digestible energy; *ME* Metabolizable energy; *NE* Net energy; *SEM* Standard error of the mean

^2^D×X is the interaction between diet and xylanase supplementation

**Table 5:** Nutrient digestibility and energy values of the oat bran and wheat bran^1^

Item^2^	Ingredient^3^				SEM	*P*-value
	Oat bran		Wheat bran			
	–	+	–	+		
ATTD, %						
Gross energy	83.0^a^	81.5^a^	63.3^c^	71.8^b^	6.3	0.045
Crude protein	83.1^a^	79.5^ab^	52.4^c^	77.5^b^	6.8	0.034
Neutral detergent fiber	73.6^a^	65.3^ab^	47.0^c^	62.8^b^	7.8	0.020
Acid detergent fiber	52.2^a^	43.8^b^	28.0^c^	52.8^a^	4.7	0.031
Organic matter	83.7^a^	83.4^a^	58.0^c^	75.0^b^	5.0	0.044
Energy utilization, %						
ME/DE	97.7	97.2	98.4	96.3	3.6	0.405
NE/ME	77.5	79.6	74.7	77.1	5.4	0.223
Energy values, MJ/kg DM						
DE	16.80^a^	16.50^a^	11.93^b^	13.53^ab^	0.72	0.122
ME	16.41^a^	16.04^a^	11.75^c^	13.02^b^	0.80	0.033
NE	12.72^a^	12.76^a^	8.77^c^	10.04^b^	0.95	0.046

^a,b,c^Values with different characters in superscripts were different (*P* < 0.05) in the same row

^1^Treatment means are reported as least squares means and are based on 6 observations per treatment

^2^The apparent total tract digestibility (ATTD) of nutrient and energy values of oat bran and wheat bran were calculated using difference method in diets containing 36% oat bran or 27% wheat bran. *DE* Digestible energy; *ME* Metabolizable energy; *NE* Net energy; *SEM* Standard error of the mean

^3^The – and + symbol represents enzyme-free dietary treatment and enzyme addition dietary treatment, respectively

### Short-chain fatty acids in feces

The total SCFA were considered as the sum of lactate, formate, acetate, propionate, isobutyrate, butyrate, isovalerate, and valerate, but the lactate and formate were not listed because of their minor concentrations (Table 6). The concentrations of SCFA were expressed in mg/kg feed DM. There were interactions (*P* < 0.05) between diet and enzyme supplementation on concentrations of acetate, propionate, butyrate, and isovalerate. In the enzyme-free dietary groups, pigs fed WB-based diets excreted more (*P* < 0.05) acetate and propionate and total SCFA in feces than those fed a basal diet. The acetate and propionate concentrations in feces were greater (*P* < 0.05) in pigs fed 27% WB diets compared with those fed 36% OB diets and a basal diet. The acetate concentrations in feces were greater (*P* < 0.05) in pigs fed 27% WB diets compared with those fed 12% WB diets. By contrast, the increase of fiber level in OB diets did not affect the acetate and propionate concentrations in fecal samples. Enzyme supplementation increased (*P* < 0.05) acetate, propionate, isobutyrate and total SCFA excretion in pigs fed 12% WB diets.

**Table 6:** Effect of dietary characteristics and xylanase addition on fecal SCFA in growing pigs^1^

Item^2^	Xylanase-free diets					Xylanase supplementation diets					SEM	*P*-value		
	Basal diet	36% OB	27% WB	15% OB	12% WB	Basal diet	36% OB	27% WB	15% OB	12% WB		Diet	Xylanase	D×X^3^
Acetate	0.68^e^	1.23^cde^	2.22^ab^	1.24^cde^	1.59^cd^	1.07^de^	1.66^bcd^	1.73^bc^	1.22^cde^	2.50^a^	0.123	< 0.001	0.002	< 0.001
Propionate	0.36^e^	0.81^cde^	1.28^ab^	0.74^cde^	0.99^bcd^	0.62^de^	1.15^abc^	0.92^bcd^	0.76^cde^	1.54^a^	0.096	< 0.001	0.015	0.002
Isobutyrate	0.04^c^	0.08^abc^	0.09^abc^	0.09^abc^	0.08^abc^	0.06^bc^	0.13^ab^	0.14^a^	0.07^abc^	0.15^a^	0.016	< 0.001	0.003	0.074
Butyrate	0.19^b^	0.35^b^	0.59^ab^	0.40^ab^	0.45^ab^	0.26^b^	0.44^ab^	0.35^b^	0.39^ab^	0.67^a^	0.065	< 0.001	0.521	0.018
Isovalerate	0.09^c^	0.17^abc^	0.24^a^	0.18^abc^	0.11^bc^	0.11^bc^	0.22^ab^	0.23^ab^	0.17^abc^	0.26^a^	0.025	< 0.001	0.009	0.024
Valerate	0.09^b^	0.20^ab^	0.17^ab^	0.23^ab^	0.15^ab^	0.15^ab^	0.28^a^	0.14^ab^	0.18^ab^	0.24^ab^	0.037	0.023	0.184	0.221
Total SCFA	1.48^e^	2.88^cde^	4.65^ab^	2.90^cd^	3.41^bcd^	2.29^de^	3.95^bc^	3.58^bcd^	2.83^cde^	5.42^a^	0.295	< 0.001	0.012	0.004

^a,b,c,d,e^Values with different characters in superscripts were different (*P* < 0.05) in the same row

^1^Treatment means are reported as least squares means and are based on 6 observations per treatment. *OB* Oat bran; *WB* Wheat bran; *SCFA* Short-chain fatty acid; *SEM* Standard error of the mean

^2^SCFA concentration (mg/g Feed DM) = SCFA concentration (mg/g feces DM) × feces DM (g)/feed DM (g)

^3^D×X is the interaction between diet and xylanase supplementation

### The richness and biodiversity of bacterial communities

**Fig. 1:**
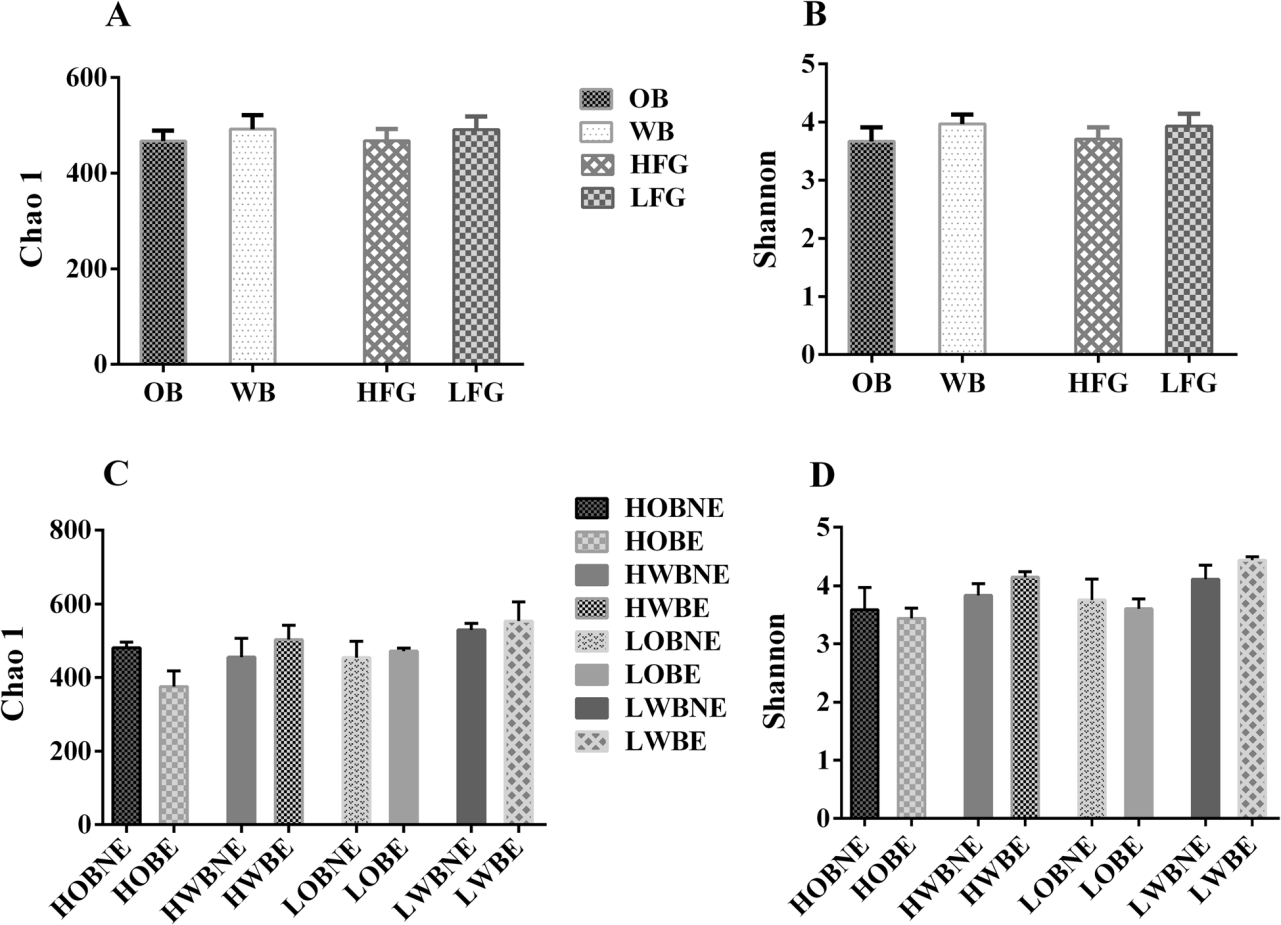
Effects of fiber type, fiber level and xylanase addition on the richness and diversity of microbial communities in growing pigs. **a** Chao 1 index of bacterial community between OB and WB and between HFG and LFG. **b** Shannon index of bacterial community between OB and WB and between HFG and LFG. **c** Chao 1 index of bacterial community in pigs fed experimental diets. **d** Shannon index of bacterial community in pigs fed experimental diets. The results were analyzed by Welch’s t-test and presented as mean values. OB, oat bran-based diets; WB, wheat bran-based diets; HFG, diet containing 36% OB and diet containing 27% WB (without enzyme); LFG, diets containing 15% OB or 12% WB (without enzyme). HOBNE, HOBE, HWBNE, HWBE, LOBNE, LOBE, LWBNE, and LWBE mean diets containing 36% OB without enzyme, 36% OB with enzyme, 27% WB without enzyme, 27% WB with enzyme, 15% OB without enzyme, 15% OB with enzyme, 12% WB without enzyme, and 12% WB with enzyme, respectively

**Fig. 2:**
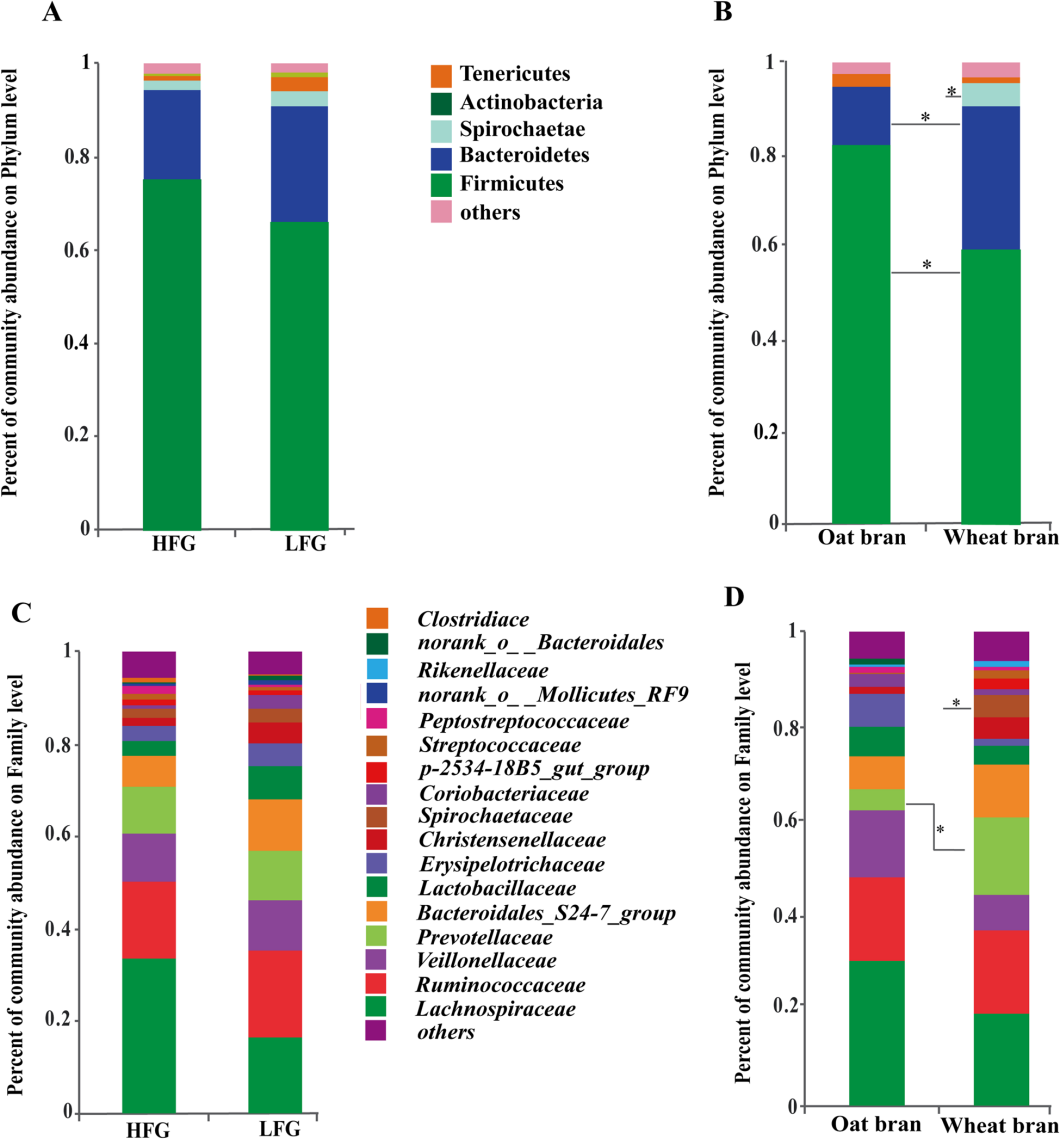
Effects of fiber level and type fecal on microbial community structure in growing pigs. Distribution of fecal bacteria on the phylum (**a**) and family (**c**) levels in pigs fed high or low-fiber diets. Distribution of fecal bacteria on the phylum (**b**) and family (**d**) levels in pigs fed OB or WB based diets. The Welch’s t test was applied to identify the differences in the relative abundance of gut microbiota between two groups, and one asterisk means *P* < 0.05. Phyla and families with proportions less than 1% are not listed. HFG, diet containing 36% oat bran or 27% wheat bran (without enzyme); LFG, diets containing 15% oat bran or 12% wheat bran (without enzyme)

The indices of Chao and Shannon at the OTU level were used to elevate bacterial richness and diversity. The two indices were not affected by fiber source, fiber level or enzyme addition (Fig. 1). The bacterial composition was presented at the phylum, family, and genus levels, respectively (Fig. 2 and Supplementary Fig. S1). Firmicutes and Bacteroidetes, were the two main phyla of bacteria in the fecal samples of growing pigs (Supplementary Fig. S1A). The predominant families in Firmicutes consisted of Ruminococcaceae, Lachnospiraceae, Veillonellaceae, and Christensenellaceae, while Prevotellaceae, Bacteroidales_S24-7_group, and Erysipelotrichaceae were the predominant families in Bacteridetes (Supplementary Fig. S1B).

**Fig. 3:**
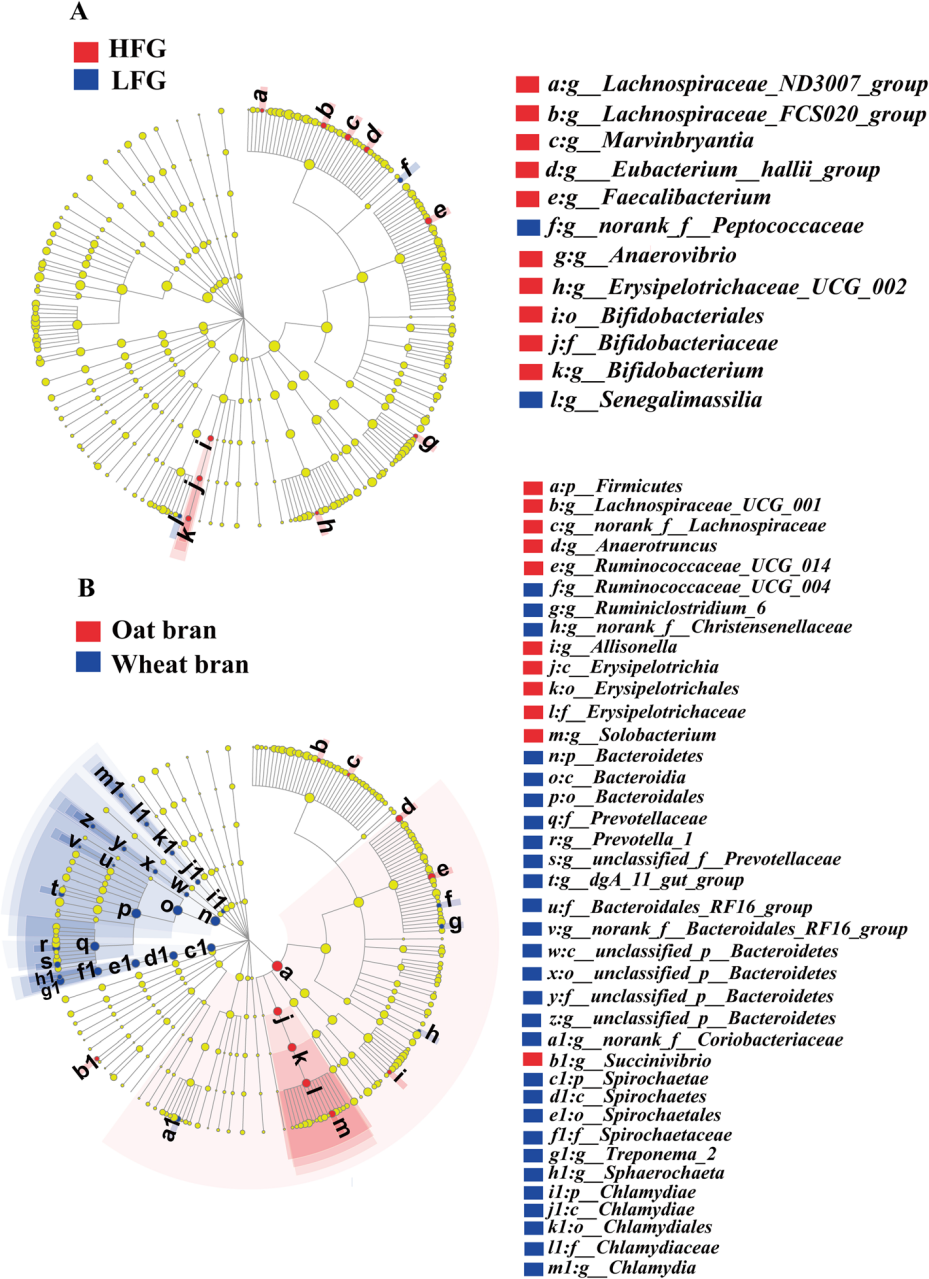
Effects of fiber level and type on differential bacteria from genus to phylum. **a** Differential bacteria (*P* < 0.05) between HFG and LFG. **b** Differential bacteria (*P* < 0.05) between oat bran and wheat bran treatments. HFG, diet containing 36% oat bran and diet containing 27% wheat bran (without enzyme); LFG, diets containing 15% oat bran or 12% wheat bran (without enzyme); oat bran, oat bran-based diets; wheat bran, wheat bran-based diets

**Fig. 4:**
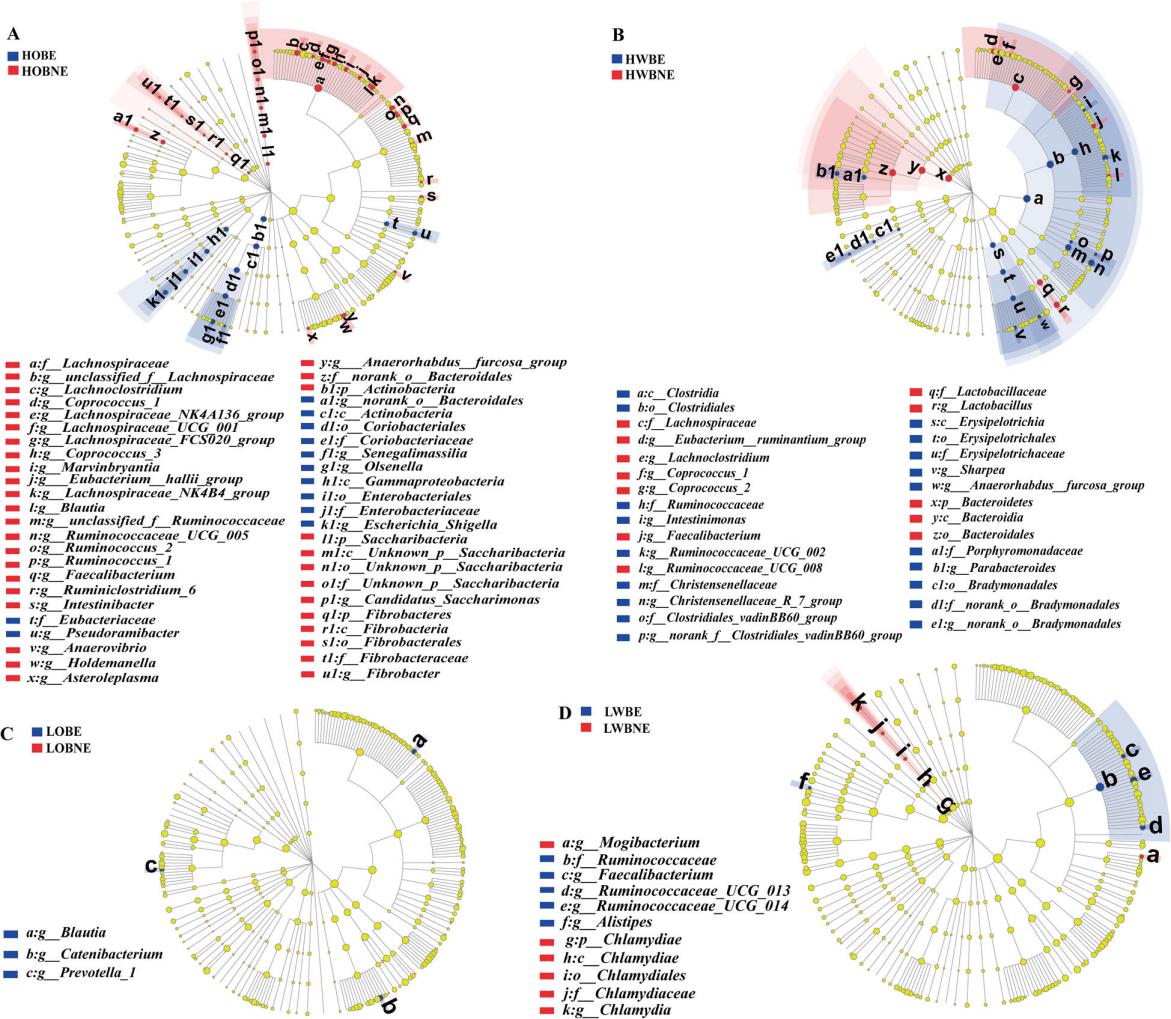
Effects of xylanase on differential bacteria from genus to phylum. **a** Differential bacteria (*P* < 0.05) between HOBE and HOBNE. **b** Differential bacteria (*P* < 0.05) between HWBE and HWBNE. **c** Differential bacteria (*P* < 0.05) between LOBE and LOBNE. **d** Differential bacteria (*P* < 0.05) between LWBNE and LWBE. HOBNE, HOBE, HWBNE, HWBE, LOBNE, LOBE, LWBNE, and LWBE mean diets containing 36% OB without enzyme, 36% OB with enzyme, 27% WB without enzyme, 27% WB with enzyme, 15% OB without enzyme 15% OB with enzyme, 12% WB without enzyme, and 12% WB with enzyme, respectively

Cladogram of LEfSe showed all differential bacteria from phylum to genus level (Fig. 3 and Fig. 4). The relative abundances of main kinds of bacteria were greater (*P* < 0.05) in high-fiber diets than those in low-fiber diets (Fig. 3a), including *Marvinbryantia, [Eubacterium]_hallii_group, Lachnospiraceae_FCS020_group* and *Lachnospiraceae_ND3007_group,* which belonged to family Lachnospiraceae, and *Bifidobacterium,* which belonged to family Bifidobacteriaceae. The abundances of *Senegalimassilia* and *norank_f__Peptococcaceae* were decreased in high-fiber diet than those in low-fiber diets (*P* < 0.05).

The abundance of *Anaerotruncus, Ruminococcaceae_UCG_014, and Solobacterium* was greater (*P* < 0.05) in pigs fed OB diets than pigs fed WB, which resulted in an increase (*P* < 0.05) in the population of phylum Firmicutes (Fig. 3b). Pigs fed the WB diets had greater (*P* < 0.05) proportion of phylum Bacteroidetes, family Prevotellaceae and order Bacteroidia compared to those fed the OB diets, which was due to increased relative abundances of *unclassified_f__Prevotellaceae, Prevotella_1, and dgA_11_gut_group* (*P* < 0.05). The increased abundance of *Succinivibrio* (*P* < 0.05) led to greater proportion of Spirochaetae (*P* < 0.05) in pigs fed WB diets compared with those fed OB diets.

When pigs were fed diets with 36% OB (Fig. 4a), enzyme addition decreased (*P* < 0.05) the relative abundances of *unclassified_f__Lachnospiraceae, Lachnospiraceae_NK4A136_group, Lachnospiraceae_FCS020_group, Coprococcus_3, Marvinbryantia, Eubacterium__hallii_group*, and *Blautia* belonging to family Lachnospiraceae. Meanwhile, xylanase addition decreased (*P* < 0.05) the relative abundances of *Ruminococcaceae_UCG_005, Ruminococcus_1, Faecalibacterium,* and *Candidatus_Saccharimonas* and increased (*P* < 0.05) the abundances of *Escherichia_Shigella* and *Olsenella*. When pigs were fed the diet with 27% WB (Fig. 4b), enzyme addition increased (*P* < 0.05) the relative abundances of *Christensenellaceae_R-7_group* and *Ruminococcaceae_UCG_002*, thereby increasing (*P* < 0.05) the abundances of families Christensenellaceae and Ruminococcaceae. Enzyme addition decreased (*P* < 0.05) the relative abundances of genrea *Eubacterium__ruminantium_group* and *Lactobacillus* and decreased (*P* < 0.05) the abundances of families Lachnospiraceae, Bacteroidales, and Lactobacillaceae when pigs were fed diet with 27% WB. When pigs were fed the diet containing 12% OB (Fig. 4c), enzyme addition improved (*P* < 0.05) the relative abundances of *Blautia*, *Catenibacterium*, and *Prevotella_1*. When pigs were fed diet with 12% WB (Fig. 4d), enzyme addition increased (*P* < 0.05) the relative abundances of *Faecalibacterium, Ruminococcaceae_UCG_013, Ruminococcaceae_UCG_014*, thereby increasing (*P* < 0.05) the abundances of family Ruminococcaceae. In addition, enzyme addition in diet with 12% WB decreased (*P* < 0.05) the abundance of *Chlamydia*.

## Discussion

The high inclusion levels of DF in 36% OB diets and 27% WB diets decreases ATTD of DM and GE compared with basal diets. The results are consistent with the previous studies that report DF negatively affects nutrient digestibility due to their anti-nutritive effects [23–25]. The differences in chemical composition of OB and WB lead to different results on nutrient digestibility, SCFA concentrations and abundance of bacteria in feces, and energy values in diets. Wheat bran contains a higher proportion of insoluble lignified fiber and is therefore less degraded than DF in OB. As a result, pigs fed 36% OB diets have greater nutrient digestibility compared with those fed 27% WB diets. Meanwhile, 27% WB diets contain lower nutrient (DM and CP) digestibility than 12% WB diets. However, nutrient digestibility is not affected by inclusion levels of OB. The results confirm DF in WB has greater negative effects on nutrient digestibility than that in OB. In addition, OB contains more starch, CP, and EE content, which is the main reason why OB has greater NE values than WB. Wheat bran has a typical NSP composition of gramineous grain by-products that mainly consists of insoluble arabinoxylan and cellulose [26], whereas OB is rich in mixed linkages β-glucan which to a high degree is soluble and insoluble arabinoxylan. In addition, only trace levels of cellulose are found in OB [7]. In the small intestine, β-glucan in OB is partly digested while arabinoxylan in OB or WB is hardly digested [7, 8]. In pigs, fiber in OB was readily fermented by bacteria along gastrointestinal tract [27]. By contrast, most of DF in WB enters hindgut to contribute to microbial fermentation and SCFA generation. Therefore, supplementation of diets with high levels WB rather than OB caused fermentation of fiber and undigested nutrients to shift from the small intestine to hindgut, thereby contributing to growth of fiber-degrading bacteria in hindgut of pigs [27, 28]. In the current study, the abundances of Firmicutes in pigs fed OB diets are greater while the abundances of Bacteroidetes in WB diets are greater. Similarly, Liu et al. [19] showed that corn bran-based diets rich in IDF (mainly insoluble arabinoxylan) increased the population of Bacteroidetes while reducing the abundances of Firmicutes. Bacteroidetes has special polysaccharide utilization loci, which generates numerous carbohydrase enzymes to cleave the linkages that existed in complex polysaccharide structures [29]. Compared with OB diets, WB diets increase the abundance of Succinivibrio which is correlated positively with propionate and butyrate concentrations in feces [30]. The Prevotellaceae are a dominant family within Bacteroidetes phylum and produces numerous enzymes, including xylanases and β-glucanases, that are reported to be positively associated with arabinoxylan degradation in the gastrointestinal tract of animals [31, 32]. *Prevotella* has a strong ability to generate acetate [33]. The greater abundances of *Prevotella* in WB groups compared to OB groups is also reported by Zhang et al. [34] who observes that digestion of NSP and xylose in ileal digesta is correlated positively with abundance of *Prevotella*. Meanwhile, the concentrations of acetate, propionate and total SCFA are greater in pigs fed 27% WB diets than those fed 36% OB diets, which is consistent with the results about fecal bacteria. Our findings support the hypothesis that differences in chemical composition for OB diets and WB diets affect the abundance of the gut bacteria and concentrations of SCFA, and is ultimately reflected in energy metabolism.

Inconsistent results regarding the effects of NSP-degrading enzyme on nutrient digestibility based on cereal by-products have been reported widely [28, 35, 36]. In the current study, the addition of enzyme improves ATTD of nutrients and NE values of diets in pigs fed 27% WB diets. By contrast, the addition of enzyme to the basal diets or OB diets does not impact the ATTD of nutrients and NE values of diets. This observation suggests that the effect of enzymes on nutrient digestibility is related to type and concentration of substrates. Hemicellulose in WB consists predominantly of xylose and arabinose residues, and the concentration of arabinoxylan in WB is double than that in OB [37]. Consequently, diets containing 27% WB have the greatest arabinoxylan concentration among all diets. According to the theory of enzymatic kinetics, the higher the substrate concentration is, the faster the enzymatic reaction within a certain concentration range [38]. Yin et al. [24] reported that enzyme supplementation had a greater effect on ATTD of nutrients in pigs fed high NSP wheat bran-based diets compared with those fed low NSP wheat or wheat flour diets. Zeng et al. [28] also reported supplementation of carbohydrase increased ATTD of DM (74.5% vs. 78.0%) and CP (75.4% vs. 79.7%) in pigs fed wheat-wheat bran (10% wheat and 20% WB) diets, but not in wheat (10% wheat) diets. It confirms that effects of enzyme are highly related to type and concentration of the substrates. Additionally, another possible explanation is that OB contains significant quantities of insoluble arabinoxylans along with soluble β-glucans [26], so the use of xylanase does not eliminate the anti-nutritional effects of β-glucans. Soluble β-glucans increase viscosity of digesta, which, in turn, prevent arabinoxylans to contact with xylanase. The explanation is confirmed by a previous study that reports that xylanase is more efficient in degrading arabinoxylan in wheat than in rye. In addition, Yin et al. [39] reported that β-glucanase or an enzyme cocktail (β-glucanase, xylanase and protease) added to barley diets (higher proportion of β-glucans) improves ATTD of NDF (60.9%, 68.1%, 66.6% for control diets, β-glucanase addition diets and cocktai addition diets, respectively) but adding xylanase alone has no effect on the ATTD of NDF although values varied greatly (60.9% vs. 67.1%). It indicates that the effects of enzyme depend on whether the type of enzyme matches the dietary composition. Furthermore, the enzyme addition not only has an impact on digestion of specific NSP but also other nutrients by improving probability for nutrients to contact with endogenous enzymes [14]. In the current study, the enzyme addition improves the NE values only for 27% WB diets. Because DF provides a negligible net amount of DE to growing pigs [40], the explanation for this result is that the enzyme supplementation improves ATTD of CP and DM, resulting in more energy sources available to pigs. Exogenous enzymes influenced the intestinal microbiota by enhancing nutrient delivery to the host, providing fermentable oligosaccharides degraded from indigestible polysaccharides, and affecting the fermentation site and viscosity and flow rate of digesta [9, 14]. In general, addition of carbohydrase enzymes influenced more bacterial abundances in pigs fed high-fiber diets compared to pigs fed low-fiber diets. The results were consistent with those of digestibility, SCFA, and energy metabolism in high-fiber diets (mainly 27% WB diets) and low-fiber diets. Zhao et al. [41] reports that SCFA concentrations in feces are positively correlated with the IDF digestibility in diets. Enzyme addition increases SCFA concentrations in feces when pigs are fed 12% WB diets, which may be attributed to increased fiber digestibility (57.5% vs. 60.7% for NDF and 56.2% vs. 60.4% for ADF). However, there is an inconsistent result for effects of enzyme on 27% WB diets. This maybe because enzyme addition hydrolyzed cell wall components, promoting the digestion of nutrients in the foregut, resulting in a decrease in the substrate available for hindgut fermentation. Enzyme supplementation decreases abundance of Lachnospiraceae, Bacteroidales, and Lactobacillaceae for 27% WB-fed pigs, which seems to support this explanation. However, comprehensive explanation is not available because SCFA concentrations in feces do not represent the SCFA production in intestinal tract.

## Conclusions

In conclusion, diets supplied by high level of OB or WB promote the growth of fiber-degrading bacteria. The differences in chemical composition between WB and OB lead to differences in nutrient digestibility and bacterial communities, which were ultimately reflected in energy metabolism. Enzyme supplementation improves nutrient digestibility as well as NE values for 27% WB but not for other diets, which indicates that effects of enzyme are determined by type and level of NSP in diets. However, the SCFA production and absorption is not measured in this study, leading to an inability to comprehensively explain the correlation between fatty acids and energy metabolism. Therefore, the effect of dietary structure on digestive properties in different intestinal segments deserves further study.

## Supplementary information

**Additional files 1: Table S1.** Chemical composition of fibrous ingredients (%, as-fed basis)^1^. **Table S2.** Effect of dietary characteristics and xylanase addition on heat production and energy retention of growing pigs^1^. **Figure S1.** Effects of xylanase on fecal microbial community structure in growing pigs.

## Data Availability

The data were shown in the main manuscript and supplemental materials.

## Abbreviations

AA: Amino acid
ADF: Acid detergent fiber
ATTD: Apparent total tract digestibility
BSNE: The basal diet without xylanase
BW: Body weight
CH_4_E: Energy lost as methane
CP: Crude protein
DE: Digestible energy
DF: Dietary fiber
DM: Dry matter
EE: Ether extract
FG: Wheat bran diets and oat bran diets without xylanase
FHP: Fasting heat production
GE: Gross energy
HFG: Diet containing 36% oat bran and diet containing 27% wheat bran (without enzyme)
HP: Heat production IDF Insoluble dietary fiber
LDA: Linear discriminant analysis effect size
LEfSe: Linear discriminant analysis effect size
LFG: Diets containing 15% oat bran or 12% wheat bran (without enzyme)
ME: Metabolizable energy
NDF: Neutral detergent fiber
NE: Net energy
NSP: Non-starch polysaccharide
OB: Oat bran
OBNE: Oat bran diets without xylanase
OUT: Operational taxonomic units
OM: Organic matter
RDP: Ribosomal database project
RE: Retained energy
RE_L_: Retained energy as lipids
RE_P_: Retained energy as protein
SCFA: Short-chain fatty acids
SDF: Soluble dietary fiber
TDF: Total dietary fiber
THP: Total heat production
UE: Gross energy of urine
WB: Wheat bran
WBNE: Wheat bran diet without xylanase
