# Supplementary material for: Oat bran and wheat bran impact net energy by shaping microbial communities and fermentation products in pigs fed diets with or without xylanase
Source: J Anim Sci Biotechnol. 2020 Oct 8;11:99. doi: 10.1186/s40104-020-00505-7 (PMC7542896; doi:10.1186/s40104-020-00505-7)

**Supplementary Table S1.** Chemical composition of fibrous ingredients (%, as-fed basis)^1^

| Item | Oat bran | Wheat bran |
| --- | --- | --- |
| Dry matter | 92.5 | 88.7 |
| Gross energy, MJ/kg | 18.7 | 16.8 |
| Crude protein | 22.0 | 15.1 |
| Starch | 28.1 | 8.2 |
| Ether extract | 7.6 | 3.2 |
| Ash | 4.7 | 6.0 |
| NDF | 30.7 | 47.8 |
| ADF | 6.6 | 14.1 |
| Hemicellulose | 24.1 | 33.7 |
| Total dietary fiber | 35.8 | 43.2 |
| Soluble dietary fiber | 15.0 | 2.7 |
| Insoluble dietary fiber | 20.8 | 40.5 |
| Insoluble non-starch polysaccharides | 15.6 | 28.7 |
| Rhamnose | 0.6 | 0.6 |
| Arabinose | 2.8 | 6.6 |
| Xylose | 4.7 | 11.0 |
| Mannose | 0.6 | 0.8 |
| Galactose | 0.4 | 0.6 |
| Glucose | 4.6 | 8.0 |
| Uronic acids | 1.9 | 1.1 |
| Soluble non-starch polysaccharides | 7.8 | 3.9 |
| Rhamnose | 0.1 | 0.1 |
| Arabinose | 0.2 | 0.6 |
| Xylose | 0.1 | 1.1 |
| Mannose | 0.1 | 0.2 |
| Galactose | 0.2 | 0.1 |
| Glucose | 6.9 | 1.6 |
| Uronic acids | 0.2 | 0.2 |
| Total non-starch polysaccharides | 23.4 | 32.6 |

^1^Data are the means of two replicates of analyzed values. NDF, neutral dietary fiber; ADF, acid detergent fiber. Hemicellulose was calculated as the difference in neutral detergent fiber and acid detergent fiber.

**Supplementary Table S2.** Effect of dietary characteristics and xylanase addition on heat production and energy retention of growing pigs^1^

|  | | Xylanase-free diets | | | | |  | | Xylanase supplementation diets | | | | |  | *P*-value | | |
| --- | --- | --- | --- | --- | --- | --- | --- | --- | --- | --- | --- | --- | --- | --- | --- | --- | --- |
| Item^2^ | Basal diet | | 36% OB | 27% WB | 15% OB | 12 % WB |  | Basal diet | | 36% OB | 27% WB | 15% OB | 12 % WB | SEM | Diet | Xylanase | D×X^3^ |
| Energy balance, kJ/(kg BW^0.6^·d) | | | |  |  |  |  |  | |  |  |  |  |  |  |  |  |
| ME intake | 2296 | | 2309 | 2179 | 2406 | 2248 |  | 2,326 | | 2447 | 2357 | 2377 | 2343 | 65 | 0.202 | 0.036 | 0.451 |
| THP | 1130 | | 1199 | 1200 | 1150 | 1181 |  | 1,138 | | 1217 | 1191 | 1219 | 1130 | 47 | 0.401 | 0.552 | 0.702 |
| Adjusted THP | 1140 | | 1204 | 1257 | 1143 | 1210 |  | 1,136 | | 1169 | 1177 | 1198 | 1122 | 51 | 0.483 | 0.473 | 0.694 |
| FHP | 771 | | 797 | 751 | 730 | 797 |  | 752 | | 802 | 802 | 817 | 769 | 51 | 0.691 | 0.929 | 0.639 |
| RE_P_ | 453 | | 492 | 418 | 464 | 443 |  | 472 | | 509 | 437 | 474 | 434 | 21 | 0.311 | 0.521 | 0.924 |
| Adjusted RE_P_ | 460 | | 498 | 453 | 459 | 461 |  | 471 | | 479 | 428 | 460 | 429 | 15 | 0.133 | 0.179 | 0.409 |
| RE_L_ | 713 | | 618 | 560 | 727 | 605 |  | 716 | | 723 | 716 | 685 | 747 | 63 | 0.515 | 0.056 | 0.172 |
| Adjusted RE_L_ | 722 | | 620 | 612 | 714 | 651 |  | 714 | | 676 | 705 | 665 | 732 | 54 | 0.661 | 0.392 | 0.274 |
| Total RE | 1166 | | 1110 | 978 | 1191 | 1048 |  | 1188 | | 1232 | 1153 | 1159 | 1181 | 70 | 0.593 | 0.062 | 0.285 |
| Adjusted RE | 1182 | | 1118 | 1065 | 1173 | 1112 |  | 1185 | | 1155 | 1133 | 1125 | 1161 | 52 | 0.895 | 0.584 | 0.386 |
| Respiratory quotient |  | |  |  |  |  |  |  | |  |  |  |  |  |  |  |  |
| Fed state | 1.06 | | 1.04 | 1.05 | 1.05 | 1.05 |  | 1.07 | | 1.04 | 1.05 | 1.06 | 1.06 | 0.01 | 0.093 | 0.109 | 0.902 |
| Fasted state | 0.81 | | 0.82 | 0.82 | 0.81 | 0.82 |  | 0.82 | | 0.82 | 0.82 | 0.82 | 0.83 | 0.02 | 0.562 | 0.672 | 0.554 |

^1^*n* = 6.

^2^OB, oat bran, WB, wheat bran; THP, total heat production; FHP, fasting heat production; RE, retained energy; RE_P_, retained energy as protein; RE_L_, retained energy as lipids. Adjusted RE_P_, RE_L_, and RE means the RE_P_, RE_L_, and RE was adjusted for a ME intake of 2,322 kJ/(kg BW^0.60^·d) (mean value for the experiment) by covariance, respectively; SEM, standard error of the mean.

^3^D×X is the interaction between diets and xylanase supplementation.

**Supplementary Fig S1. Effects of xylanase on fecal microbial community structure in growing pigs.** (A) Microbial community barplot on phylum level with the abundance higher than 1%. (B) Microbial community barplot on family level with the proportion higher than 1%. (C) Microbial community heatmap of top 30 bacteria on genus level. Differential bacteria between two groups were analyzed using the Welch’s *t* test, and one asterisk means *P* < 0.05. HOBNE, HOBE, HWBNE, HWBE, LOBNE, LOBE, LWBNE, and LWBE mean diets containing 36% OB without enzyme, 36% OB with enzyme, 27% WB without enzyme, 27% WB with enzyme, 15% OB without enzyme, 15% OB with enzyme, 12% WB without enzyme, and 12% WB with enzyme, respectively.


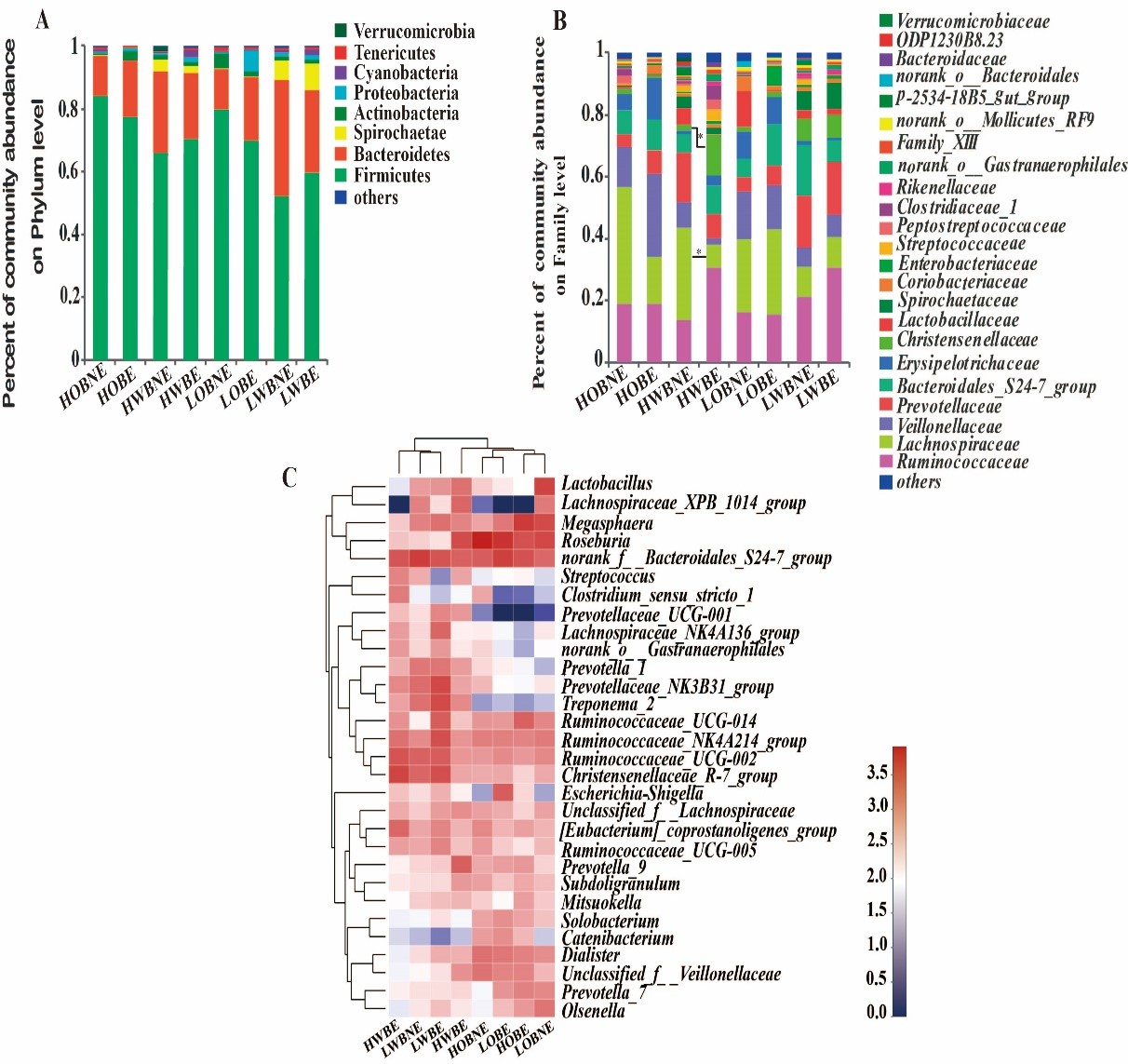

Supplement: Supplementary file 1 — Additional files 1: Table S1. Chemical composition of fibrous ingredients (%, as-fed basis)1. Table S2. Effect of dietary characteristics and xylanase addition on heat production and energy retention of growing pigs1. Figure S1. Effects of xylanase on fecal microbial community structure in growing pigs. [file 40104_2020_505_MOESM1_ESM.docx]
